# Supplementary material for: MEMS-tunable dielectric metasurface lens
Source: Nat Commun. 2018 Feb 23;9:812. doi: 10.1038/s41467-018-03155-6 (PMC5824825; doi:10.1038/s41467-018-03155-6)
Supplement: Supplementary file 1 — Supplementary Information [file 41467_2018_3155_MOESM1_ESM.pdf]

Arbabi et. al. “MEMS-tunable dielectric metasurface lens”

SUPPLEMENTARY FIGURES

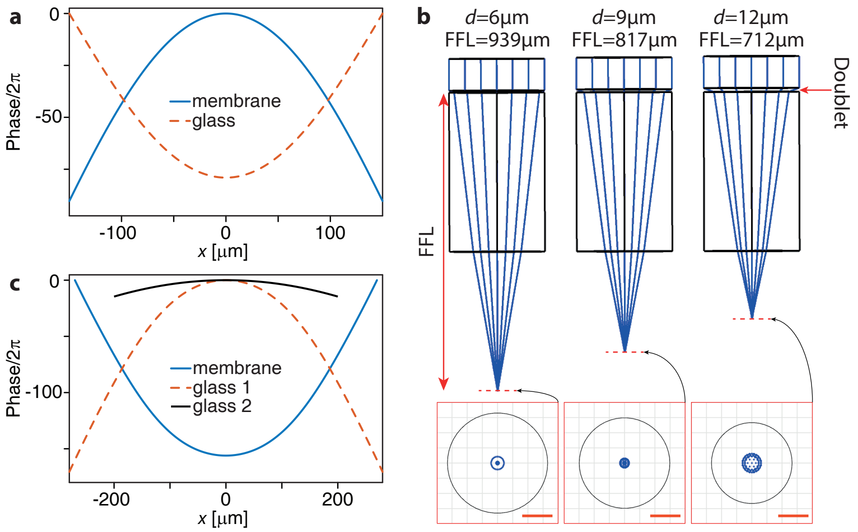

Supplementary Figure 1. **Phase profiles and ray optics simulations.** (**a**) Optimized phase profiles for the fabricated doublet metasurfaces. (**b**) Ray optics simulation results for the doublet in three different configurations. The scale bars are 2 μm. FFL: Front focal length. (**c**) Phase profiles for the three elements of the microscope shown in Fig 5.

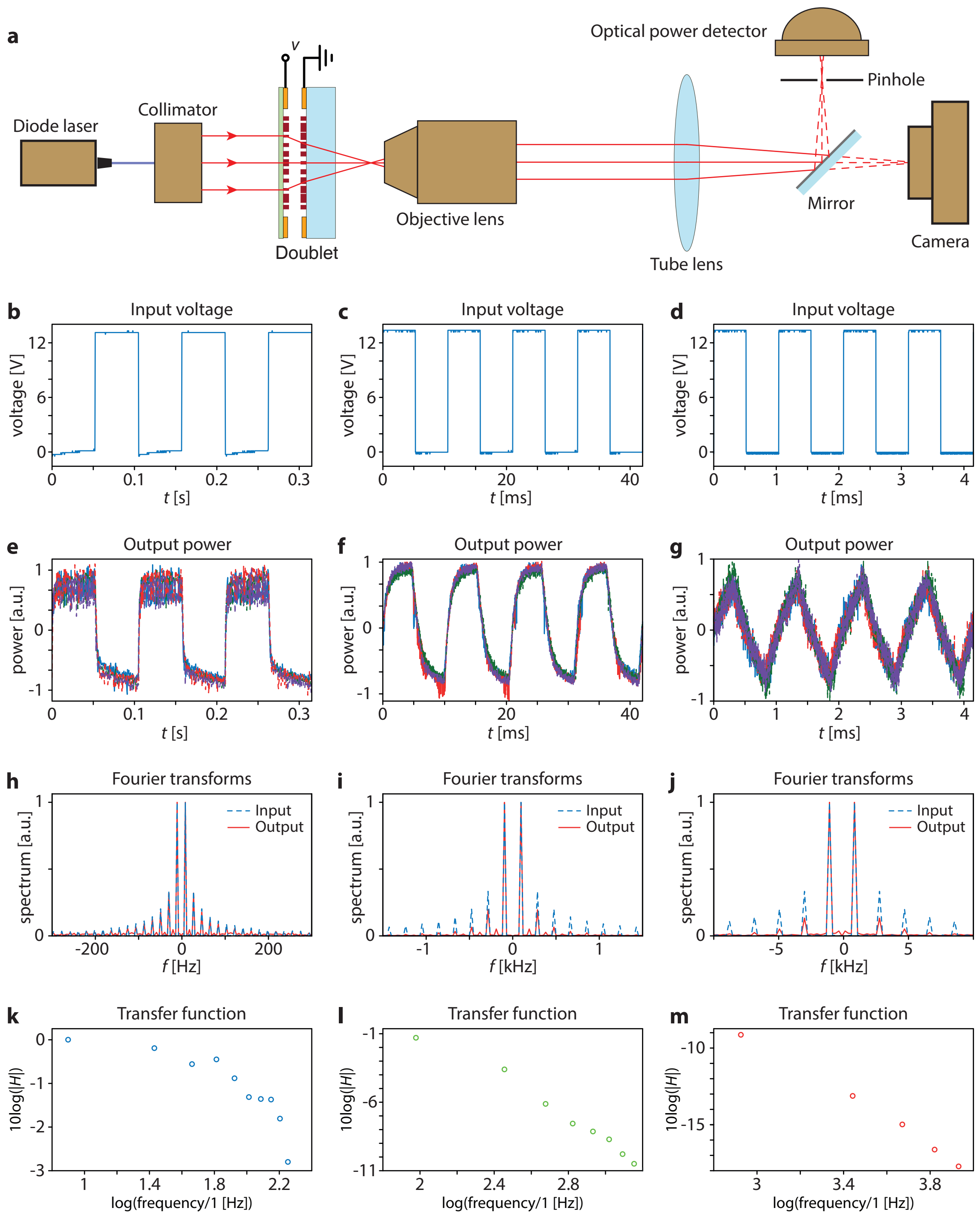

Supplementary Figure 2. **Measurement setup and raw frequency measurement results.** (**a**) Measurement setup used to characterize the doublet. The mirror, pinhole and optical power detector are used for measurement of frequency response and efficiency. (**b**-**d**) Input voltage waveforms for different base frequencies. (**e**-**g**) Measured output powers for corresponding input voltages b-d. (**h**-**j**) Fourier transforms of the mean measured output voltages. (**k**-**m**) The calculated pieces of the transfer function over different frequency bands. See Supplementary Note 1 for details of the measurement procedure.

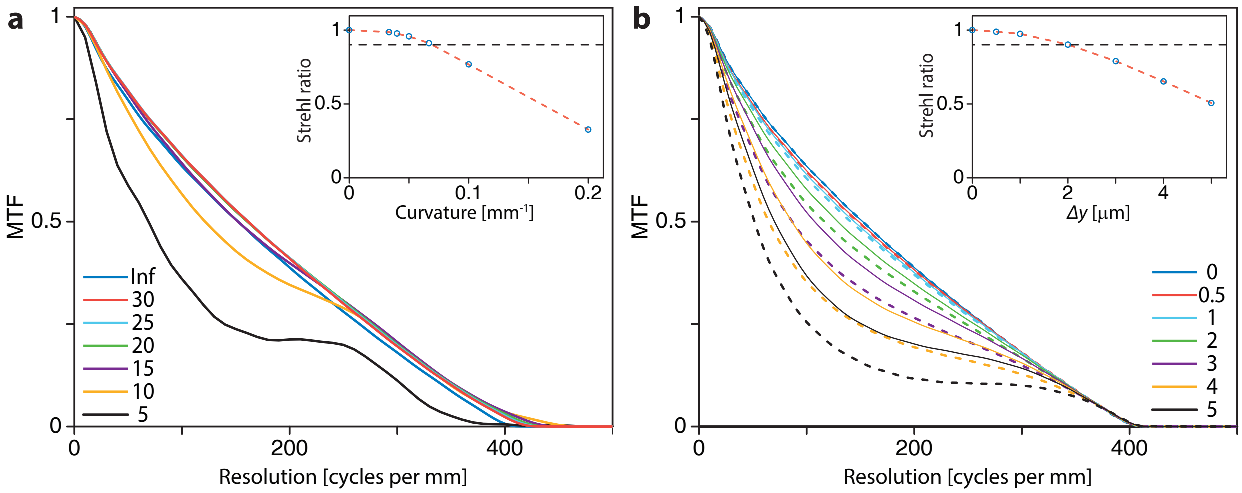

Supplementary Figure 3. **Aberrations resulting from membrane bending and lens misalignment.** (**a**) On-axis modulation transfer function (MTF) of the doublet plotted for various radii of curvature (the numbers are in millimeters). Inset: the calculated Strehl ratio plotted versus curvature (i.e., one over radius). The red dashed line is an eye-guide, and the black dashed line corresponds to a Strehl ratio of 0.9. (**b**) On-axis modulation transfer function of the doublet plotted for various transverse displacements between the lenses (the numbers are in micrometers). For each color, the solid line is the sagittal plane, and the dashed line is the tangential plane. Inset: the calculated Strehl ratio plotted versus displacement. The red dashed line is an eye-guide, and the black dashed line corresponds to a Strehl ratio of 0.9.

SUPPLEMENTARY TABLES

Supplementary Table 1. Optimized phase profile coefficients for the fabricated doublet.

Element	R_0 [μm]	a_2	a_4	a_6	a_8	a_{10}	a_{12}	a_{14}
Moving lens	150	-6.479$\times10^2$	5.430$\times10^1$	8.997$\times10^1$	-1.532$\times10^2$	1.557$\times10^2$	-8.498$\times10^1$	1.871$\times10^1$
Stationary lens	150	5.835$\times10^2$	-4.717$\times10^1$	-1.317$\times10^2$	2.324$\times10^2$	-2.201$\times10^2$	7.967$\times10^1$	3.145$\times10^0$

Supplementary Table 2. Optimized phase profile coefficients for the simulated triplet microscope.

Element	R_0 [μm]	a_2	a_4	a_6	a_8	a_{10}
Moving lens	270	8.657$\times10^2$	7.828$\times10^2$	-1.240$\times10^3$	7.004$\times10^2$	-1.281$\times10^2$
Stationary lens 1	280	-9.800 $\times10^2$	-7.474$\times10^2$	1.280$\times10^3$	-8.157$\times10^2$	1.921$\times10^2$
Stationary lens 2	200	-9.354$\times10^1$	1.675$\times10^0$	5.765$\times10^{-1}$	-6.854$\times10^{-1}$	0.2783$\times10^{-1}$

SUPPLEMENTARY NOTE 1: FREQUENCY RESPONSE MEASUREMENT

The frequency response of the doublet is defined as the ratio of the membrane movement upon application of an alternating voltage at frequency *f*, to the membrane movement upon application of the same DC voltage. To measure this response we used the setup schematically shown in Supplementary Fig. 2**a** (including the mirror, a 50-μm pinhole, and a power detector). Square shaped pulse trains at different frequencies were applied to the contacts (Supplementary Figs. 2**b**-2**d**). Modulation of the optical power passing through the pinhole is also measured with the power detector connected to an oscilloscope (Supplementary Figs. 2**e**-2**g**). To maximize the modulation of the optical power passing through the pinhole, the objective was adjusted to image a plane a few microns away from the focal spot of the doublet. The optical power was measured in four different intervals (plotted in the figures with different colors), and their average was used as the output to reduce noise. The input voltage and output powers were then Fourier transformed, resulting in the spectra shown in Supplementary Figs. 2**h**-2**j**. The transfer function for each input frequency band (i.e., for each of the Supplementary Figs. 2**b**-2**d** inputs) can be calculated via dividing the output and input spectra at the harmonic frequencies (Supplementary Figs. 2**k**-2**m**). We chose the base harmonic frequencies (i.e., 10 Hz, 100 Hz, and 1 kHz) so that there is an overlap in the bandwidths where the transfer function is calculated for each input. Using this overlap, we can cascade the transfer functions to find the frequency response plotted in Fig. 3**e** of the manuscript. We should note that there are inherent approximations in the method used here for finding the frequency response. First, the membrane displacement is a nonlinear function of the applied voltage. Second, the change in the focal distance with the membrane movement is weakly nonlinear. Third, the optical power passing through the pinhole is a nonlinear function of the focal distance change. Assuming that all changes are small compared to their respective rest values, we can linearize all these functions, and find the transfer functions. Nevertheless, traces of the nonlinearities could be seen in the excitation of output harmonics that are not present in the input (Supplementary Figs. 2**h**-2**j** inputs). The fact that these new harmonics have small amplitudes compared to linearly excited ones, and the good agreement between the resulting frequency response and the over-damped fit indicate that the linearization approximation is valid.
